# Polyamine transporter potABCD is required for virulence of encapsulated but not nonencapsulated *Streptococcus pneumoniae*

**DOI:** 10.1371/journal.pone.0179159

**Published:** 2017-06-06

**Authors:** Haley R. Pipkins, Jessica L. Bradshaw, Lance E. Keller, Edwin Swiatlo, Larry S. McDaniel

**Affiliations:** Department of Microbiology and Immunology, University of Mississippi Medical Center, Jackson, Mississippi, United States of America; Instituto Butantan, BRAZIL

## Abstract

*Streptococcus pneumoniae* is commonly found in the human nasopharynx and is the causative agent of multiple diseases. Since invasive pneumococcal infections are associated with encapsulated pneumococci, the capsular polysaccharide is the target of licensed pneumococcal vaccines. However, there is an increasing distribution of non-vaccine serotypes, as well as nonencapsulated *S*. *pneumoniae* (NESp). Both encapsulated and nonencapsulated pneumococci possess the polyamine oligo-transport operon (*potABCD*). Previous research has shown inactivation of the pot operon in encapsulated pneumococci alters protein expression and leads to a significant reduction in pneumococcal murine colonization, but the role of the pot operon in NESp is unknown. Here, we demonstrate deletion of *potD* from the NESp NCC1 strain MNZ67 does impact expression of the key proteins pneumolysin and PspK, but it does not inhibit murine colonization. Additionally, we show the absence of *potD* significantly increases biofilm production, both in vitro and in vivo. In a chinchilla model of otitis media (OM), the absence of *potD* does not significantly affect MNZ67 virulence, but it does significantly reduce the pathogenesis of the virulent encapsulated strain TIGR4 (serotype 4). Deletion of *potD* also significantly reduced persistence of TIGR4 in the lungs but increased persistence of PIP01 in the lungs. We conclude the pot operon is important for the regulation of protein expression and biofilm formation in both encapsulated and NCC1 nonencapsulated *Streptococcus pneumoniae*. However, in contrast to encapsulated pneumococcal strains, polyamine acquisition via the pot operon is not required for MNZ67 murine colonization, persistence in the lungs, or full virulence in a model of OM. Therefore, NESp virulence regulation needs to be further established to identify potential NESp therapeutic targets.

## Introduction

*Streptococcus pneumoniae* (the pneumococcus) is a gram positive coccus that colonizes the nasopharynx of humans [1]. Although colonization is typically asymptomatic, the organism can gain access to normally sterile sites and cause illnesses such as pneumonia, otitis media (OM), sinusitis, bacteremia, and meningitis [2]. In young children, the elderly, and immunocompromised patients, these pneumococcal infections can lead to serious complications or even death [3]. Pneumococcal strains can be typed using either multilocus sequence typing (MLST) or by serotyping of the polysaccharide capsule [4]. Strains that lack serological evidence of capsule expression are divided into two nonencapsulated *S*. *pneumoniae* (NESp) groups [5]. Group I includes NESp that have a mutated, dysfunctional variant of the characteristic capsule polysaccharide biosynthetic (*cps*) locus, while group II includes NESp whose *cps* genes are replaced by novel genes that code for distinct pneumococcal proteins [6,7].

Currently, there are two licensed pneumococcal vaccines used in the United States: Prevnar13 and Pneumovax [8]. Because most invasive pneumococcal infections are associated with encapsulated pneumococci, these vaccines target the capsular polysaccharide of 13 or 23 invasive disease-associated capsular serotypes, respectively [9]. However, non-invasive pneumococcal disease rates remain high even with vaccine implementation. For example, middle ear infections are the most frequent condition for which antibiotic treatment is prescribed to children under the age of five in the U.S., and *S*. *pneumoniae* is the leading cause of acute bacterial OM [10–12]. This inefficiency is likely due to selective pressure against the pneumococcal capsule resulting in capsular alterations in vaccine serotypes and increased colonization by non-vaccine serotypes and NESp [13–15]. A recent study in Japan analyzed pneumococcal isolates taken from the nasopharynges of children with OM, and of those isolates 6.4% were NESp [16]. The distribution of NESp will likely continue to escalate as selective pressure is placed on encapsulated strains. Since current vaccines do not protect against NESp strains, it is important to identify new targets that will protect against both encapsulated and nonencapsulated pneumococci.

All sequenced pneumococcal isolates, whether encapsulated or NESp, possess the polyamine oligo-transport operon (*potABCD*) [17]. A proposed description of each operon component is as follows: PotA is an ATP binding protein that couples polyamine translocation with ATP hydrolysis, PotBC compose an α-helical transmembrane polypeptides with hydrophobic domains, and PotD is a membrane protein responsible for binding extracellular polyamines [18]. Polyamines are cationic organic compounds that are found ubiquitously in nature and are essential for normal cell growth of both eukaryotic and prokaryotic cells [19,20]. The role of polyamine acquisition and biosynthesis in bacterial growth and virulence has recently been investigated, and it has been determined that these pathways are integral to bacterial fitness and pathogenesis [21–23]. Additionally, all four genes of the operon are essential for operon function, as mutation or deletion of any one of the genes nullifies activity [24].

Analyses have shown the deletion of *potD* in encapsulated pneumococcal isolates significantly reduces their ability to colonize and cause disease [25]. The pot operon and the transport of polyamines also appears to be necessary for regulation of certain encapsulated pneumococcal virulence factors, such as pneumolysin and capsule [17,18]. Furthermore, immunization with recombinant PotD both reduces pneumococcal nasopharyngeal colonization and prevents systemic pneumococcal infection in a mouse model [26–28]. However, the effects of a *potD* mutant in NESp have not yet been established. If *potD* deletion reduces NESp virulence as efficiently as it does in encapsulated pneumococci, then PotD could potentially be a therapeutic target against both types of pneumococci. Since the *pot* operon has thus far been identified in all sequenced pneumococci, increased prevalence of non-vaccine strains could be controlled by targeting PotD. The rationale for this study was therefore to determine the role of the pot operon in the virulence and pathogenesis of NESp subgroup NCC1.

## Materials and methods

### Bacterial strains and growth conditions

Descriptions of bacterial strains used for this work are listed in Table 1. All pneumococcal strains were grown at 37°C in 5% CO_2_ in Todd-Hewitt medium with 0.5% yeast extract (THY) or on blood agar base supplemented with 5% sheep blood (BA). Mutant strains were grown in the presence of the appropriate antibiotic selection as indicated in Table 1. For all experiments, strains were grown to an OD_600_ of 0.3. Bacterial lysates were prepared by suspending 1.5 ml pelleted bacteria in 150 μl lysis buffer (0.01% sodium dodecyl sulfate, 0.1% sodium deoxycholate, and 0.015 M sodium citrate), incubating the suspension at 37°C for 30 minutes, and then diluting the mixture with 150 μl phosphate-buffered saline (PBS). Plasmid DNA was extracted from *Escherichia coli* using a plasmid miniprep kit following manufacturer’s protocols (Qiagen).

**Table 1:** Description of strains used for the studies described in this manuscript.

Strain	Strain Description
MNZ67	PspK^+^ Group II NESp (Subgroup NCC1); source of *potD*
PIP01	MNZ67 isogenic Δ*potD* mutant (Kan^R^)
PIP02	PIP01 transformed with pNE1:: *potD* (Spec^R^)
TIGR4 (T4)	Virulent encapsulated pneumococcal isolate (serotype 4)
T4 *ΔpotD*	TIGR4 isogenic Δ*potD* mutant (Erm^R^)
DAR831*ΔAR4*	Encapsulated pneumococcus; source of Kan^R^ cassette (Serotype 12F)

### PCR amplification

Polymerase chain reaction (PCR) amplification was used to isolate a kanamycin resistance cassette from pneumococcal strain DAR831Δ*AR4* (donated by Dr. Ashley Robinson at The Univeristy of Mississippi Medical Center) (primers 5 and 6). PCR was also used to amplify a 750 bp segment upstream of *potD* (primers 1 and 2) and a 700 bp segment downstream from *potD* (primers 3 and 4) from the NESp isolate MNZ67. These three fragments were combined using sequence overlap extension (SOE), and the full-length fragment was amplified using PCR (primers 1 and 4) [29]. PCR was additionally used to amplify *potD* from MNZ67 (primers 7 and 8), which was cloned into the spectinomycin resistant plasmid pNE1 to generate pNE1::*potD*. Products less than 1 kb were amplified using GoTaq DNA Polymerase (Promega) and products greater than 1 kb were amplified using Phusion High Fidelity DNA Polymerase (Thermo-Scientific). Cycling parameters recommended by the manufacturers and an annealing temp of 55° were used for the amplification process, and size of amplified products was verified by electrophoresis on an 0.8% agarose gel with EtBr staining. All primers used for these studies are listed in Table 2.

**Table 2:** Names and sequences of primers used in these studies.

Reference Number	Primer Name	Primer Sequence (5’ to 3’)
1	potD upstream F	TGG GAC TGG TCT TTC TGG TCC TCT
2	potD upstream R	CAT TAT CCA TTA AAA ATC AAC GGG CTT GCT CCT CCT TCT CAC GA
3	potD downstream F	TAC CTA TAA TTC ACC AAA AAT AAA AGA G ATA AAC GTA AGA AGA CGA ATC AG
4	potD downstream R	AACTTGTCATTCTCTTGTTCTTATGCAATTGTA
5	Kan F	CCG TTG ATT TTT AAT GGA TAA TG
6	Kan R	TTT TAT TTT TGG TGA ATT ATA GGT A
7	potD F	GCG GCA TGC GAG AGG AGA GAA A ATG AAA AAA ATC TAT TCA TTT TTA GCA GG
8	potD R	GCG CTG CAG CTA CTT CCG ATA CAT TTT AAA CTG
9	PspK F RT	CTGTGAAAGCAGAGATGGCA
10	PspK R RT	CCTCAGCAACCTTGCTCTTT
11	GyrA F RT	GCGAGCTCTTCCTGATGTTC
12	GyrA R RT	GGGTCACACCCAATTCATTC

### Construction of mutant strain

An isogenic *potD* deletion mutant (PIP01) of MNZ67 was constructed by allelic replacement of the *potD* gene with a kanamycin resistance cassette flanked by the upstream and downstream regions of *potD* (see PCR amplification for details). MNZ67 grown in competence media (THY containing .02% CaCl, .04% glucose, and .2% BSA) was activated with 200 ng of each of competence stimulating peptide 1 and 2 (CSP 1 and CSP 2) for 12 min followed by transformation with 500 ng of the kanamycin SOE construct for 3 hours. Selection for *ΔpotD* mutants was done by plating on BAP containing 500 μg/ml kanamycin. Complementation of *potD* in PIP01 was achieved by transforming PIP01 with pNE1::*potD* under previously described conditions, generating PIP02. Successful transformants were selected by plating on BA containing 300 μg/ml spectinomycin.

### Real time PCR

Bacterial cultures were grown to an OD_600_ of 0.3. Cells were pelleted by centrifugation and enzymatically lysed with 200 μl TE buffer (10 mM Tris, 1 mM EDTA) containing 3 mg/ml lysozyme. Bacterial RNA was purified using a Qiagen RNeasy Mini kit, and collected RNA was treated with RQ1 DNase (Promega) following manufacturer’s protocol. RNA concentrations were determined using a Qubit 2.0 fluorometer. RT-qPCR was performed using 200 ng total RNA, a Luna Universal One-Step RT-qPCR Kit (New England Biolabs), and gene specific primers. Primers 9 and 10 were used for *pspK* and primers 11 and 12 were used for *gyrA*, which served as an internal control. A no template sample was used as a negative control. Samples were analyzed using an iCycler iQ Real-Time PCR Detection System (Biorad), and the ΔΔCT method was used to calculate the fold-change in gene expression.

### Protein standardization

A standard bicinchoninic acid (BCA) assay protocol [30] was used to quantify total protein concentration of bacterial lysates. Protein concentrations were determined by reading samples on an xMark microplate spectrophotometer (BioRad) at OD_570_. Lysates were standardized to equivalent protein amounts by diluting with sterile PBS.

### Hemolysis assay

Standardized bacterial lysates were serially diluted in 96-well round bottom plates containing 100 μl DTT buffer (10 ml PBS, 0.01g BSA, 0.015g DTT) in each well. Hemolysis was determined by adding 50 μl of PBS containing 1% sheep red blood cells (RBCs) to each well, followed by incubation at 37°C in 5% CO_2_ for 30 minutes. Triton X-114 served as the positive control, and PBS was used as the negative control. The plate was centrifuged at 500 x g for 5 minutes. 100 μl of supernatant was removed from each well and transferred to a 96-well flat bottom plate. The plate was read at OD_450_ on an xMark microplate spectrophotometer (BioRad), and percent lysis was normalized to lysis by detergent set at 100%. Samples were plated in triplicate and three independent experiments were performed.

### Production of pneumolysin (Ply) and pneumococcal surface protein K (PspK)

#### ELISA

Protein levels of both Ply and PspK were determined using a standard indirect ELISA protocol. Blocking buffer used consisted of PBST and 1% BSA. Polyclonal rabbit anti-Ply or rabbit anti-PspK serum was used as the primary antibody. Secondary antibody for all assays was a polyclonal, biotinylated donkey anti-rabbit IgG combined with streptavidin-conjugated alkaline phosphatase. Protein was visualized by addition of P-nitrophenyl phosphate and plates were read at OD_405_ on an xMark microplate spectrophotometer (BioRad). Protein levels were normalized to wild type (WT) MNZ67 set at 100% expression. All samples were analyzed in triplicate and three independent experiments were performed.

#### Flow cytometry

PspK was further examined using flow cytometry. Approximately 10^7^ mid-log phase bacteria were pelleted by centrifugation, suspended in PBS containing rabbit anti-PspK serum, and incubated for 30 minutes at 37°C [31]. Pneumococcal cells were fluorescently labeled by incubation for 30 minutes at room temperature with biotinylated polyclonal donkey anti-rabbit IgG combined with streptavidin-conjugated Alexa Flour 488. Bacteria were washed three times in sterile PBS after each incubation step and surface expressed PspK was analyzed using a Becton Dickinson flow cytometer with gate set to 25,000 bacteria.

### Biofilm formation

Biofilm production was determined by seeding a 24-well plate with 10^5^ CFU of bacteria in 1 ml of THY containing 8 units/ml catalase and 10% horse serum. The plate was incubated for 24 hours at 37°C in 5% CO_2_. The media was removed and each well was stained with 350 μl 0.1% crystal violet for 30 minutes at room temperature. The excess crystal violet was carefully removed and the remaining crystal violet was solubilized in 1 ml 100% ethanol for 10 minutes with shaking. The plate was read at OD_630_ on an xMark microplate spectrophotometer (BioRad). All samples were assayed in triplicate and experiments were performed three independent times.

### Epithelial cell adhesion and invasion

Human A549 (ATCC^®^ CCL-185^™^ retrieved from Dr. Stephen Stray at University of Mississippi Medical Center) and Detroit 562 (ATCC^®^ CCL-138^™^ retrieved from Dr. Justin Thornton at Mississippi State University) epithelial cells were grown to 95% confluency in Eagle’s minimal essential medium (EMEM) supplemented with 10% fetal calf serum (FCS) and containing 100 μg/ml penicillin, 100 μg/ml amphotericin B, and 50 μg/ml streptomycin. Epithelial cells were cultured in 24 well plates at 37°C in 5% CO_2_. For adhesion and invasion assays, epithelial cells were rinsed 3 times in sterile PBS and incubated with 1x10^7^ bacteria suspended in 1 ml of EMEM without antibiotics. The plates were incubated for 30 minutes for adherence or 2 hours for invasion and then washed 3 times with sterile PBS. For adhesion, cells were trypsinized with 100 μl 0.25% trypsin-EDTA, and the total volume was brought up to 1 ml by adding 900 μl PBS. For invasion, cells were incubated for an additional hour in 1 ml of EMEM containing 100 μg/ml penicillin, 100 μg/ml amphotericin B, and 50 μg/ml streptomycin. Cells were washed 3 times in PBS, detached using 100 μl 0.25% trypsin-EDTA, and then lysed in 100 μl 0.0125% Tritin X-100. All samples were plated on BA to enumerate bacteria. Three independent experiments were performed and all samples were done in triplicate.

### Murine nasopharyngeal colonization

Six to eight week old C57BL/6 mice (n = 5) were briefly anesthetized with isoflurane and intranasally inoculated with 1x10^7^ CFU pneumococci suspended in 10 μl sterile PBS. Mice were euthanized 5 days post infection by cervical dislocation, and the nasopharyngeal passage was washed with 200 μl sterile PBS. The skull was denuded, and the nasopharyngeal tissues and bullae were independently collected and homogenized in 200 μl sterile PBS. Samples were plated on BA to enumerate CFU. Animal studies performed were in accordance with protocols approved by the Institutional Animal Care and Use Committee of the University of Mississippi Medical Center. The physical condition of the mice was monitored daily by licensed veterinarians, and all measures to minimize suffering during handling and euthanasia were taken.

### Murine pneumonia

Adult C57BL/6 mice (n = 5) of were anesthetized by intraperitoneal injection of 500 μl of Avertin (2.5% amylene hydrate solution containing 6.25 mg tribromoethanol). Mice were intratracheally inoculated with 1x10^7^ CFU pneumococci and were euthanized by cervical dislocation two days post infection. The lungs were collected and homogenized in 2 ml sterile PBS. Samples were plated on blood agar to enumerate CFU. Animal studies performed were in accordance with protocols approved by the Institutional Animal Care and Use Committee of the University of Mississippi Medical Center. The physical condition of the mice was monitored daily by licensed veterinarians, and all measures to minimize suffering during handling and euthanasia were taken.

### Chinchilla otitis media

Young adult chinchillas (*Chinchilla lanigera*, 400–500 g body weight) were inoculated in each bulla via transbullar injection with 1x10^7^ CFU pneumococci suspended in 100 μl PBS containing 0.04% gelatin. Prior to infection, an otoscope was used to examine the animals’ tympanic membranes. Chinchillas were euthanized 4 days post infection in a CO_2_ chamber, and tympanic pathology was scored through otoscopic examination as follows: 0 = none, 1 = inflammation, 2 = effusion, and 3 = tympanic rupture. The skin was removed from the top of the head, and the middle ear was accessed by cutting a small hole in the top of the bulla. Visible biofilm formation was scored as follows: 0 = none, 1 = surface formation, 2 = traverses bulla, and 3 = traverses bulla with thickening. Exudate was collected and each bulla was washed with 1 ml sterile PBS. Whole bullae were removed and homogenized in 10 ml PBS. All samples were plated on BA to enumerate CFU. Animal studies performed were in accordance with protocols approved by the Institutional Animal Care and Use Committee of the University of Mississippi Medical Center. The chinchillas’ physical conditions were monitored daily by licensed veterinarians, and all measures to minimize suffering during handling and euthanasia were taken.

## Statistical analysis

Data was compared using a Student's *t* test with Prism 5 software (GraphPad Software, Inc., San Diego, CA).

## Results

### Ply activity and production

**Fig 1:**
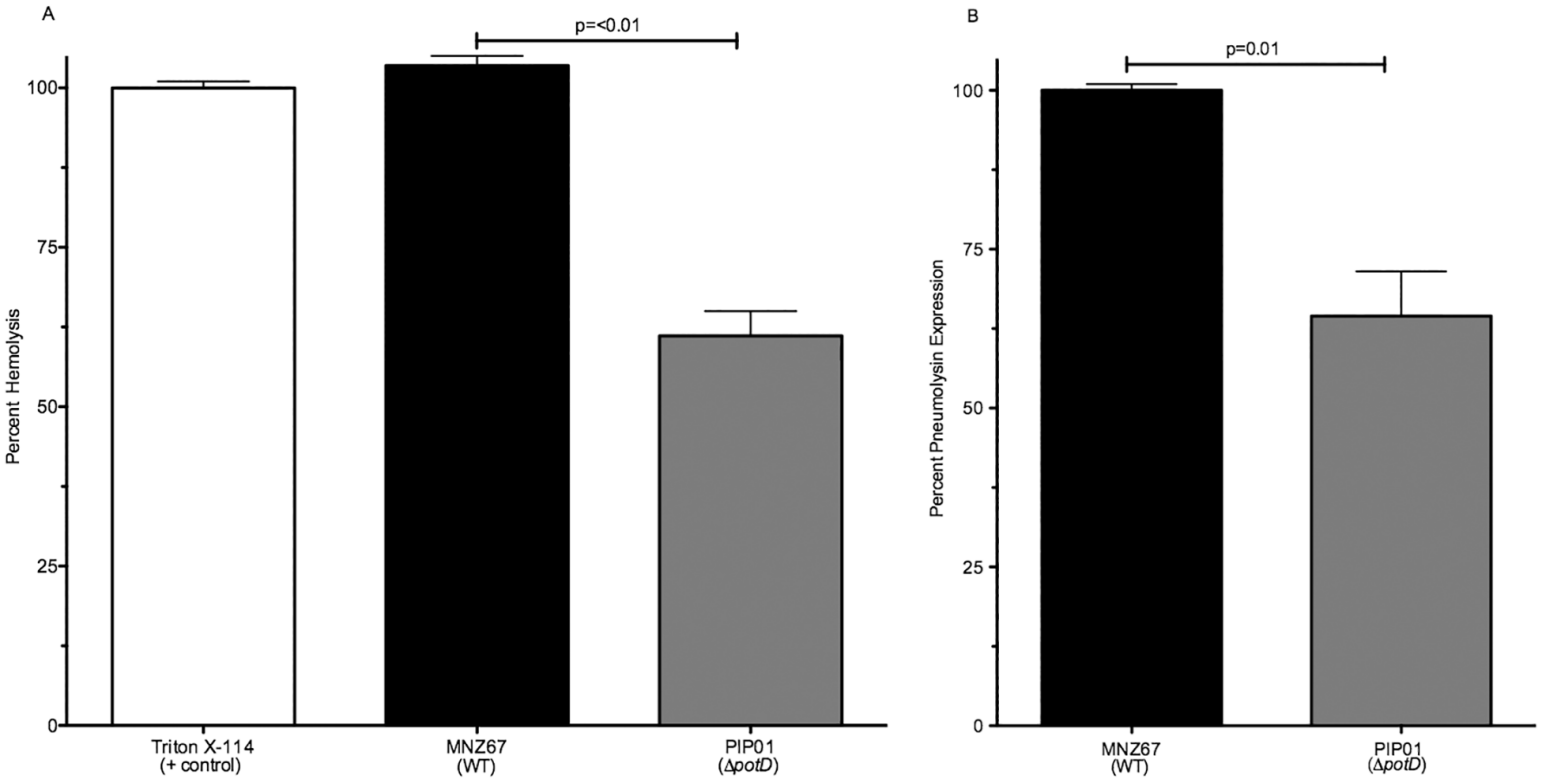
Ply activity and expression. Effects of *potD* deletion on hemolytic activity were assessed via hemolysis assay, in which pneumococcal lysates were incubated with sheep RBCs and lysis was monitored. Lysis by Triton X-114 was set at 100%. Effects on Ply synthesis were determined using a standard indirect ELISA, in which WT values were set at 100%. Deletion of *potD* from MNZ67 significantly reduced hemolytic potential (A). A significant reduction in Ply production was also observed upon deletion of *potD* (B). All samples were analyzed in triplicate and experiments were performed at least 3 times. Error bars denote standard error of the mean.

We examined the effects of *potD* deletion on the synthesis and function of the pneumococcal pore-forming toxin Ply. Hemolysis assays were performed to examine the differences in hemolytic potential between MNZ67 and PIP01. A significant reduction in RBC lysis was observed by PIP01, whereas WT MNZ67 lysed the RBCs as levels equivalent to the detergent control set at 100% (Fig 1A). To confirm the reduction in hemolytic potential was due to lower production of Ply, an ELISA was performed to establish Ply concentration. PIP01 bacterial lysates had significantly lower levels of Ply than WT MNZ67 (Fig 1B). Together, these results indicate Ply synthesis is reduced in the absence of a functional pot operon and correlate with data obtained from similar experiments using T4.

### PspK analysis

**Fig 2:**
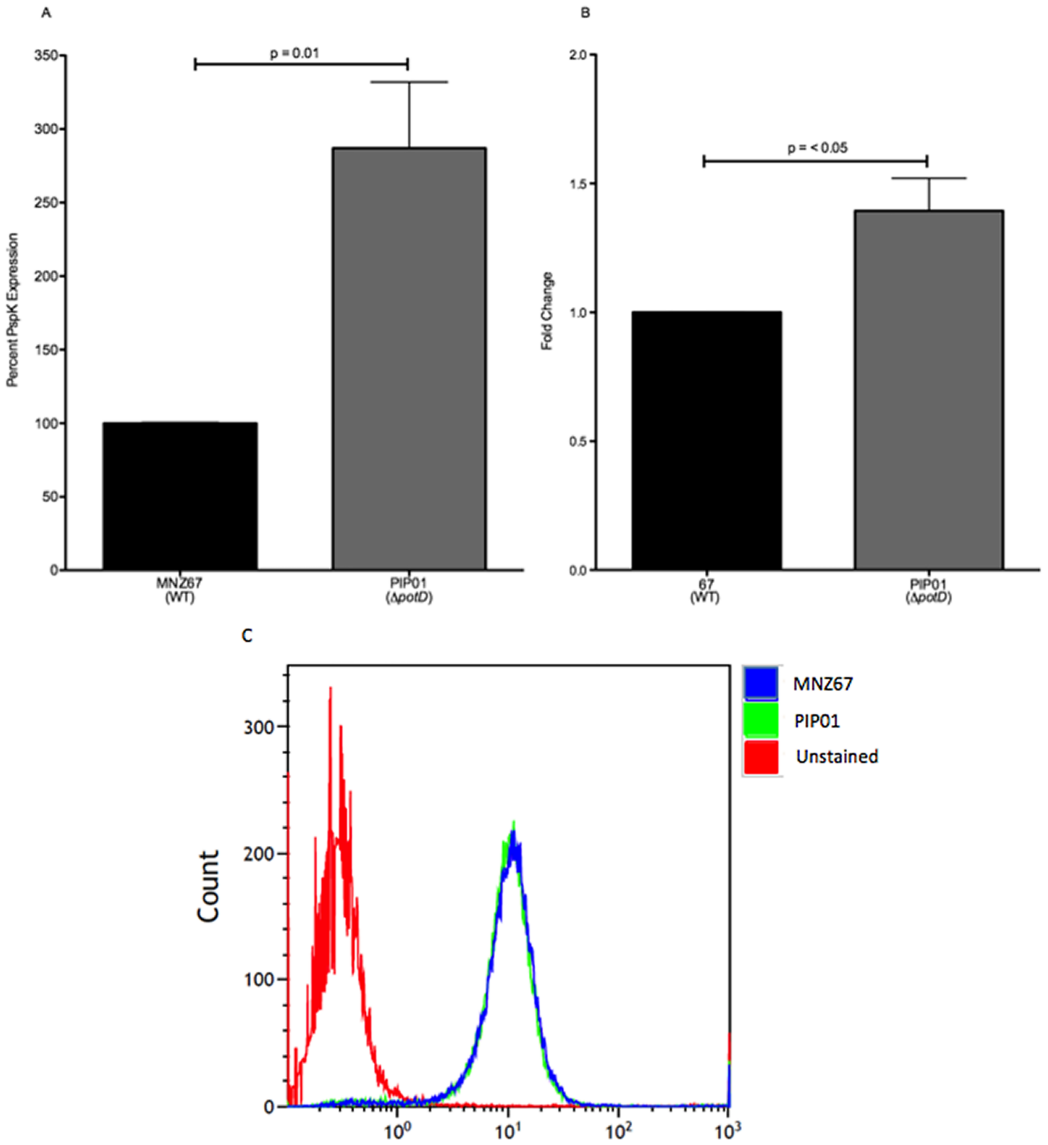
PspK analysis. PspK was quantified in pneumococcal lysates by an indirect ELISA, and *pspK* expression was determined via RT-qPCR. Surface-expressed PspK was assessed by flow cytometry. Significantly more PspK was produced in *potD* mutant PIP01 when compared to wild type MNZ67 (A). Additionally, significantly higher *pspK* expression was seen in PIP01 (B). Although increased PspK was observed, no difference was seen in surface associated PspK (C). The ELISA was performed at least three times, and each sample was analyzed in triplicate. RT-qPCR was performed three independent times and data was analyzed using the ΔΔCT method. Flow cytometry was performed twice. Error bars denote standard error of the mean.

PspK has thus far only been found in a subset of group II NESp designated NCC1, in which *pspK* replaces capsule synthesis genes [5,32]. PspK is known to increase epithelial cell adhesion and has been implicated in increased nasopharyngeal colonization, as well as enhanced acute OM [31,33]. We examined the effects of *potD* deletion on PspK production in the NESp MNZ67 using an ELISA to determine relative PspK concentrations. In comparison to MNZ67, PIP01 had significantly higher concentrations of PspK (Fig 2A). To confirm these results, we also utilized RT-qPCR to analyze *pspK* expression. In correlation with our ELISA results, we observed a significant fold change in *pspK* in PIP01 (Fig 2B). Since PspK is a surface protein, we wanted to determine if the increased PspK observed in PIP01 was actually present on the surface of viable pneumococci. The amount of surface-associated PspK was assessed by flow cytometry. Interestingly, there was not a significant difference in fluorescence intensity, signifying there was no difference in surface expressed PspK (Fig 2C). Taken together, these results indicate the absence of *potD* impacts the production of PspK.

### Complementation of *potD* expression

**Fig 3:**
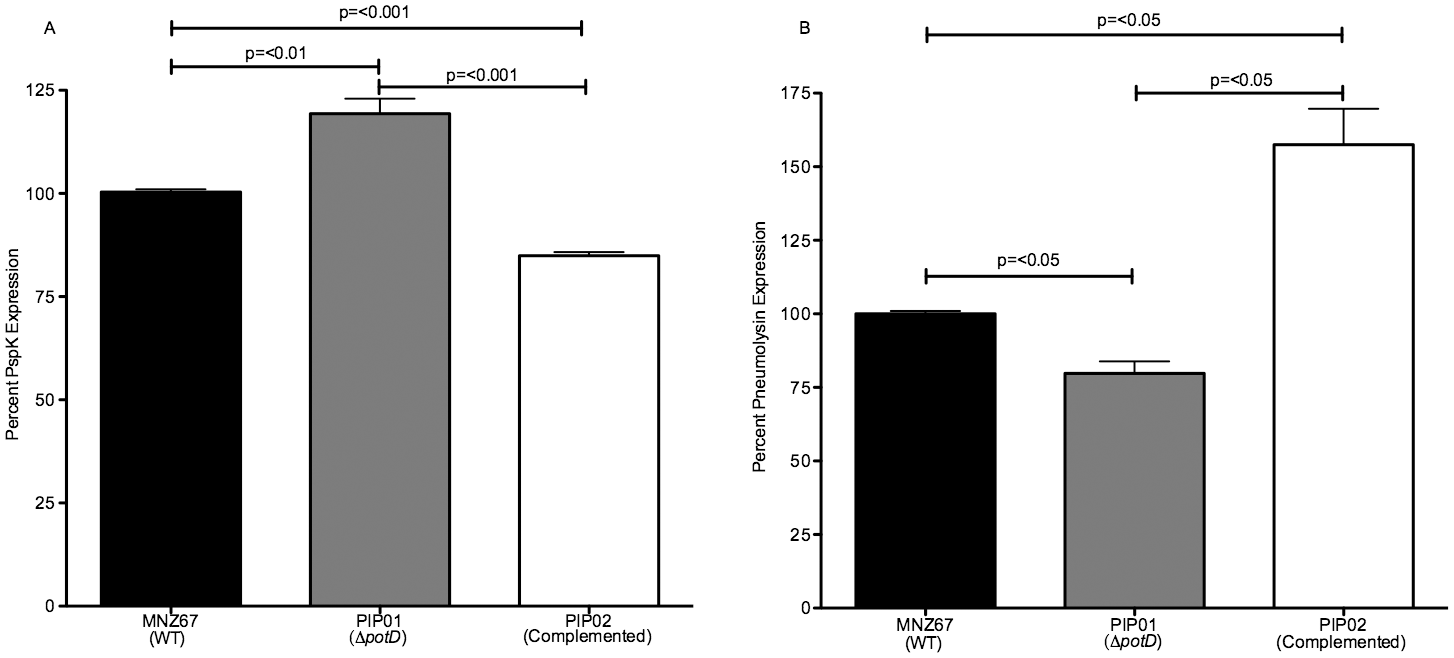
Restoration of protein synthesis in PIP01 after complementation of *potD*. PIP01 was transformed with pNE1::*potD* to produce PIP02. An indirect ELISA was used to determine effects on PspK and pneumolysin production. PspK was reduced to levels significantly less than wild type after *potD* complementation (A), while pneumolysin was increased to levels significantly higher than wild type after complementation (B). All samples were analyzed in triplicate and experiments were performed at least 3 times. Error bars denote standard error of the mean.

To validate results, *potD* was restored in PIP01, and production of Ply and PspK was analyzed via ELISA. Expression of *potD* was complemented into PIP01 via transformation with pNE1::*potD* to generate the strain PIP02. Whereas deletion of *potD* resulted in increased PspK expression and decreased Ply expression (Figs 1 and 2), restoration of *potD* resulted in decreased PspK expression (Fig 3A) and increased Ply expression (Fig 3B). Furthermore, Ply levels were significantly greater than WT, while PspK levels were significantly lower than WT (Fig 3).

### Epithelial cell adhesion and invasion

**Fig 4:**
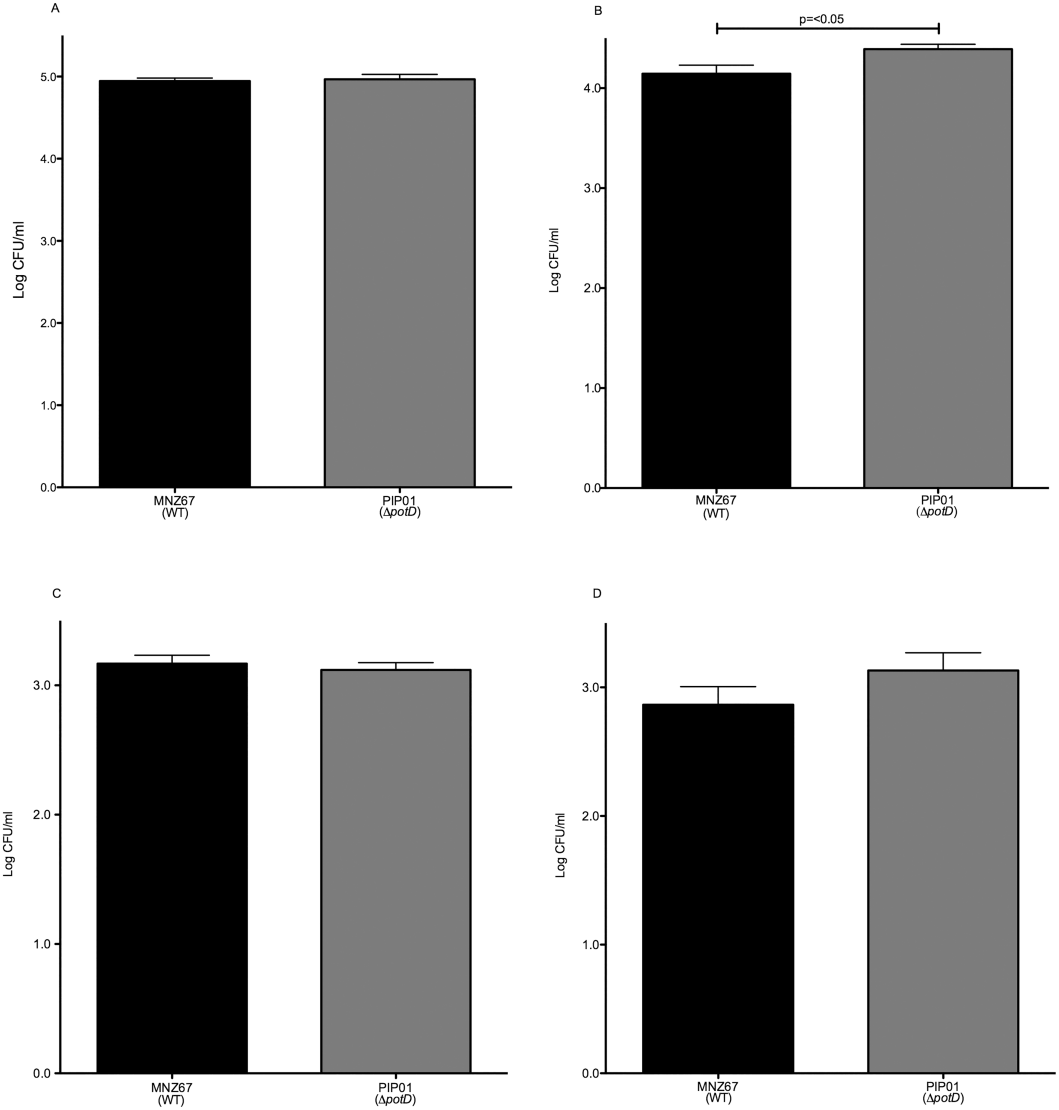
Epithelial cell adhesion and invasion. Changes in epithelial cell adhesion and invasion were assessed using adhesion/invasion assays with either Detroit 562 (pharyngeal) or A549 (lung) human epithelial cells. Epithelial cells were incubated with 10^7^ pneumococci and adhered or internalized pneumococci were enumerated on BA. Adhesion to Detroit 562 was not significantly affected by the absence of *potD* in MNZ67 (A), but adhesion to A549 cells was significantly increased in the absence of *potD* (B). A significant difference was not observed between MNZ67 and PIP01 when examining Detroit 562 or A549 cell invasion (C and D). All samples were analyzed in triplicate and experiments were performed at least 3 times. Error bars denote standard error of the mean.

Adhesion and invasion of epithelial cells is a key component of pneumococcal pathogenesis. Since an increase in the adhesive protein PspK was observed in PIP01, we analyzed whether or not adhesion to human epithelial cells was also increased. Adhesion and invasion assays were performed using two different human-derived epithelial cell lines: the pulmonary cell line A549 and the pharyngeal cell line Detroit 562. For adhesion to Detroit 562 cells, there was no discernable difference between MNZ67 and PIP01 (Fig 4A). In contrast, there was a significant increase in adhesion to A549 cells by PIP01 (Fig 4B). Epithelial cell invasion was not significantly altered when using Detroit 562 cells (Fig 4C) or A549 cells (Fig 4D).

### Biofilm production

**Fig 5:**
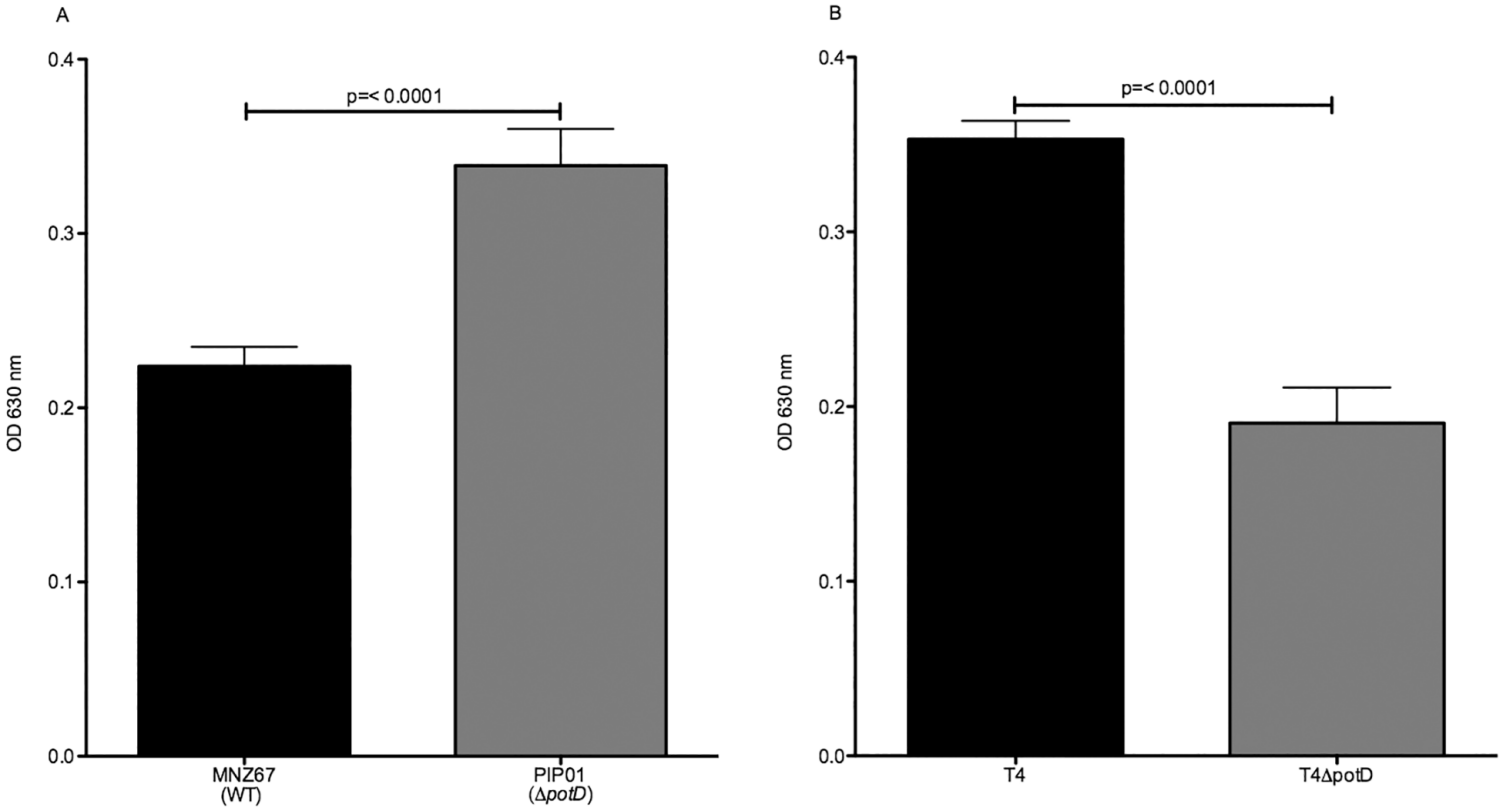
Differential effects on biofilm production. Effects of *potD* deletion on biofilm production were determined using a biofilm assay, in which biofilm formation was assessed 24 hours after seeding 1x10^5^ pneumococci in a 24 well plate. Data is presented as OD_630_. NESp PIP01 formed significantly more biofilm than WT MNZ67 (A), while T4Δ*potD* produced significantly less biofilm than WT T4 (B). Samples were analyzed in triplicate and experiments were performed at least three times. Error bars denote standard error of the mean.

It has been shown polyamine acquisition and biosynthesis play an integral role in biofilm formation of certain bacterial species [34,35]. We therefore sought to establish if polyamine transport via the pot operon was necessary for biofilm production in pneumococci and to determine if there were variances between encapsulated pneumococci and NCC1 NESp. Biofilm assays were utilized to assess biofilm formation, and results showed significantly more biofilm was formed by PIP01 in comparison to WT MNZ67 (Fig 5A). In contrast, biofilm assays using encapsulated strain T4 and a T4Δ*potD* showed biofilm formation was significantly lower in TIGR4Δ*potD* (Fig 5B). Thus, polyamine acquisition appears to differentially affect encapsulated pneumococci and NESp progression to biofilm.

### Murine colonization and ascension

**Fig 6:**
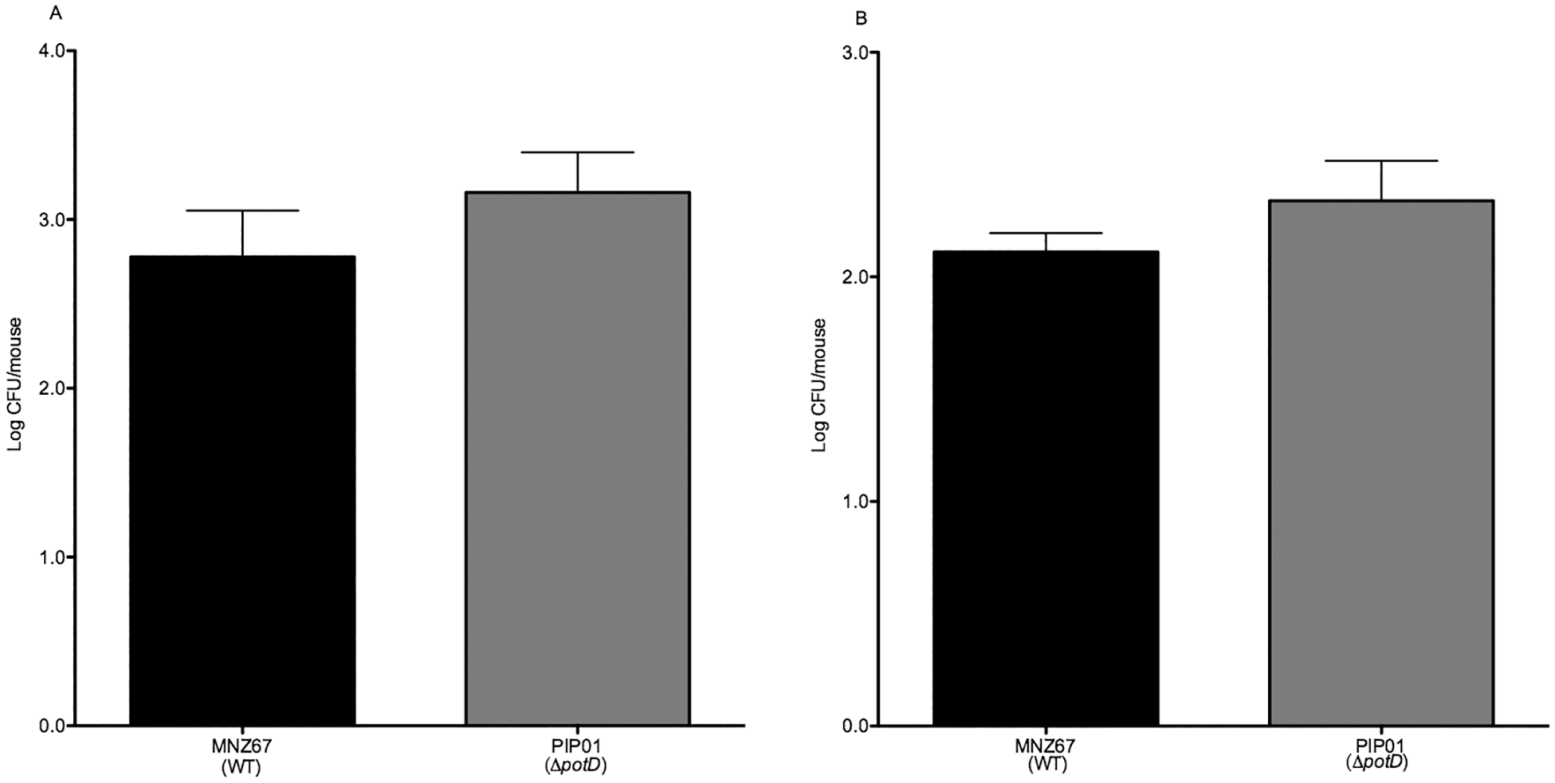
Murine nasopharyngeal colonization and ascension to the middle ear. Murine colonization and ascension were determined five days post infection after an intranasal challenge with 10^7^ pneumococci. Changes in colonization (A) and ascension (B) in the absence of *potD* were not significantly different. Five mice were infected with each strain, and two independent experiments were performed. Error bars denote standard error of the mean.

Deletion of the *pot* operon in encapsulated pneumococcal strain T4 severely attenuates its ability to colonize the nasopharynx of a mouse [17]. However, NESp Δ*potD* mutant PIP01 expresses higher levels of the adhesive protein PspK and shows increased biofilm formation (Figs 2, 3, 4, and 5A), factors that aid in colonization. We therefore investigated if the absence of *potD* significantly affects MNZ67 nasopharyngeal colonization and ascension into the middle ear in a mouse model. Interestingly, CFU recovered from the nasopharynxes and middle ears of mice infected with PIP01 did not significantly differ from those infected with MNZ67 (Fig 6).

### Chinchilla otitis media

**Fig 7:**
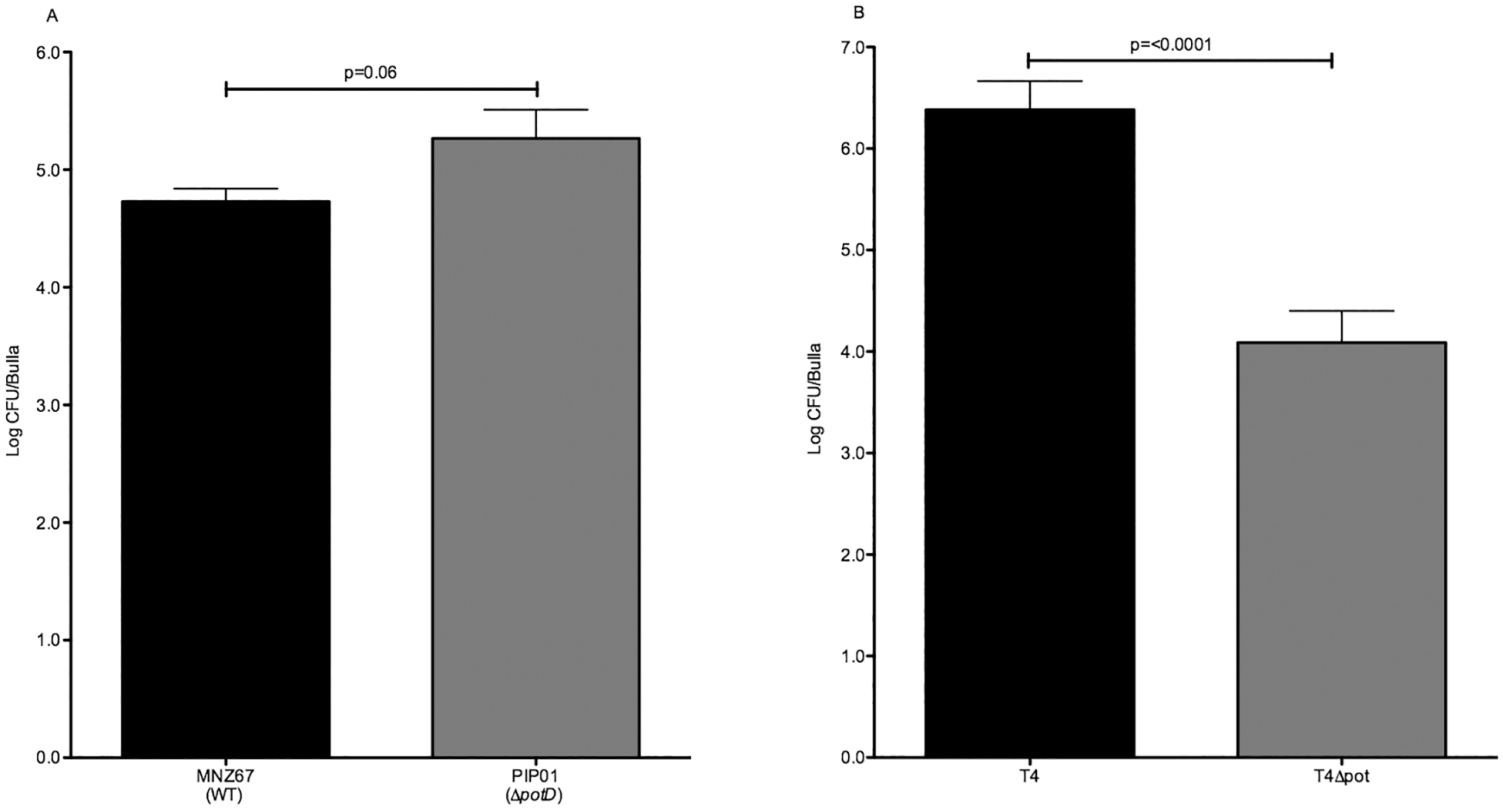
Chinchilla acute OM. Experimental OM was assessed using a chinchilla model. Animals were infected via transbullar injection and were euthanized 4 days post infection. Pneumococci recovered from chinchilla bullae did not significantly differ between WT MNZ67 and PIP01 (A). Significantly less CFU were recovered from chinchillas infected with T4Δ*potD* in comparison to wild type T4 (B). For each strain, 3 chinchillas were infected in both ears, yielding 6 individual infections. Experiments were performed twice and error bars denote standard error of the mean.

We examined the effects of *potD* deletion on pneumococcal pathogenesis during OM using a chinchilla model. Although there was no significant difference in CFU recovered from the bullae of chinchillas infected with PIP01 in comparison to those infected with MNZ67, a strong trend towards increased CFU was observed (Fig 7A). Additionally, PIP01 infected chinchillas had higher otic and biofilm scores in comparison to WT MNZ67 (Table 3). In contrast, the absence of a functional pot operon significantly reduced OM caused by T4. This included a reduction in pathology and biofilm formation (Table 3), as well as significantly fewer CFU recovered from the bullae of chinchillas infected with T4 Δ*potD* (Fig 7B). These results further illustrate the pot operon has contrasting effects on encapsulated pneumococci and NESp.

**Table 3:** OM tympanic pathology scores and biofilm scores 4 days post infection.

Strain	*Otic Score ± SEM	^#^Biofilm Score ± SEM
MNZ67	1.5 ± 0.33	1.0 ± 0.66
PIP01	2	1.75 ± 0.28
T4	2.2 ± 0.21	2.5 ± 0.24
T4 Δ*potD*	1.0 ± 0.33	0.71± 0.75

* 0 = none, 1 = inflammation, 2 = effusion, and 3 = tympanic rupture

^#^ 0 = none, 1 = surface formation, 2 = traverses bulla, and 3 = traverses bulla with thickening

### Murine pneumonia

**Fig 8:**
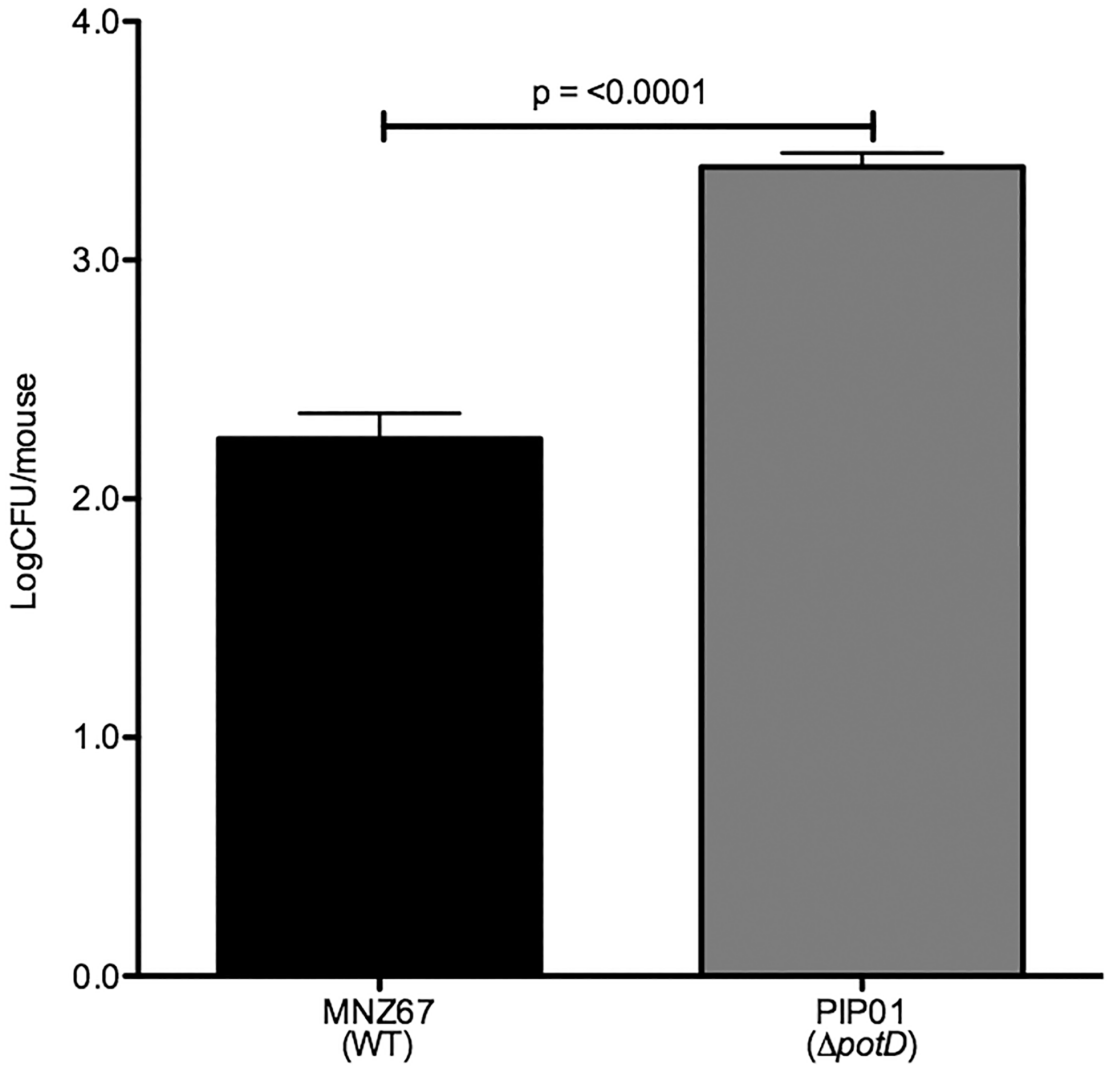
Persistence in murine lungs. The lungs of intratracheally infected mice were collected 2 days post infection, and the ability of MNZ67 and PIP01 to persist in the lungs was determined by enumerating CFU. Significantly more pneumococci were recovered from mice infected with PIP01. Five mice were infected with each strain. Error bars denote standard error of the mean.

Ware et al. demonstrated disruption of the *pot* operon significantly attenuates virulence of encapsulated strains during a pulmonary infection [25]. We investigated the ability of PIP01 to persist within the lungs of a mouse to determine if the *pot* operon is required for NESp pulmonary infections. After a two day infection period, significantly more pneumococci were recovered from the lungs of mice infected with PIP01 in comparison to those infected with MNZ67 (Fig 8). Furthermore, all five mice infected with PIP01 had bacteria in the lungs after 2 days, whereas no bacteria were recovered from the lungs of two of the mice infected with MNZ67.

## Discussion

The *pot* operon has been identified in all sequenced pneumococcal isolates [17]. Inactivation of the *pot* operon attenuates virulence of encapsulated pneumococcal strains [15,25,36], thus making the *pot* operon an intriguing target for pneumococcal treatment and prevention. However, the challenge of finding a target that truly conveys protection against all pneumococci, and not just encapsulated strains, remains an obstacle. Because research targeting the *pot* operon of encapsulated strains proved promising as a disease preventative, we aimed to evaluate the involvement of the *pot* operon in NESp virulence. Attenuation of NESp virulence after *potABCD* inactivation would confirm the *pot* operon is important for both types of pneumococci and would further validate its potential as an all-inclusive pneumococcal therapeutic. Since *potABCD* is highly conserved amongst pneumococcal isolates [17], we hypothesized it would have conserved effects on pneumococcal virulence as well. However, here we show inactivating the *pot* operon in the NESp isolate MNZ67 produces results dissimilar to those obtained using a Δ*pot* encapsulated strain. The only data that correlated with previous encapsulated studies was effects on the pore-forming toxin Ply (Fig 1). Ply is an important virulence factor of *S*. *pneumoniae* and has been shown to greatly impact the ability of the organism to cause infection [37,38]. Proteomic analysis of the virulent pneumococcal isolate T4 showed the T4-derived mutant lacking a functional pot operon had reduced expression of Ply [17]. We observed reduced lysis of RBCs when *potD* was deleted from MNZ67. Our quantitative ELISA results confirmed the loss of hemolytic potential observed in our hemolysis assay was from a decrease in Ply production. Therefore, it appears polyamine acquisition does serve a similar function in relation to regulation of this specific protein.

In MNZ67, the *cps* genes are replaced by *pspK* [32]. Examining PspK expression not only further elucidated the effects of *potD* deletion on pneumococcal protein expression, but it also allowed us to investigate the effects on a protein unique to NESp. Previous studies noted a decrease in capsule synthesis proteins when *potABCD* was deleted from T4 [17]. In contrast, we noted increased expression of PspK in MNZ67 Δ*potD* mutant, PIP01 (Fig 2). Interestingly, although *cps* and *pspK* genes occupy the same locus within the different strains, polyamine transport appears to positively regulate capsule production but negatively regulate *pspK* expression. Taken together with our Ply data and previous research involving encapsulated strains, we conclude polyamine acquisition via the pot operon is important for protein regulation in both encapsulated and nonencapsulated pneumococci. However, this appears to have strain and gene dependent variances.

To confirm *potD* deletion was responsible for changes in protein expression, we complemented *potD* in PIP01 and determined if protein levels were returned to those of wild type. When *potD* was restored in PIP01, not only was Ply increased and PspK decreased, but these changes were significantly higher or lower than WT (Fig 3). Because *potD* was complemented into PIP01 via transformation with the plasmid pNE1, these significant changes are likely due to increased copy number of *potD*. This data further indicates the involvement of polyamine acquisition in NESp protein expression regulation, as increased presence of PotD significantly alters protein levels.

We established PspK was increased in PIP01 by analyzing cell lysates via ELISA. Because PspK is a surface protein, we also wanted to determine if the increased PspK observed in PIP01 was actually expressed on the surface, or if the PspK detected by the ELISA was intracellular protein released into the environment after cell lysis. Flow cytometric analysis showed no discernable difference in surface bound PspK (Fig 2C). We therefore hypothesize these variances in expression and surface bound analyses are due to two possible explanations. The first explanation being higher levels of PspK were produced in PIP01, but it was kept within the cell and released during cell lysis; this could be because the cells already have the maximum amount of PspK on their surfaces. The second explanation is that PIP01 is actually expressing more PspK than MNZ67 on the cell surface. Due to steric hindrance, however, the antibody cannot access, and therefore cannot bind, the excess proteins, thus skewing flow cytometry results. To discern which was accurate, we looked for functionality of the increased protein.

PspK is an adhesive surface protein known to aid in adhesion to human epithelia. This adhesiveness propagates nasopharyngeal colonization and increases persistence during infection [31,33]. Our studies showed PIP01 to have increased PspK expression but no difference in surface bound PspK, which led us to investigate if PIP01 exhibited any changes in epithelial cell adhesion and invasion. We showed PIP01 had increased adhesion to human A549 pulmonary epithelial cells in vitro (Fig 4B). This data, combined with our data showing PIP01 to have increased persistence in the lungs of a mouse, led us to conclude the increased PspK being produced in PIP01 is functional and surface bound. If PspK were truncated or kept within the cell, it would not aid in adherence. However, increased PspK expression did not alter adhesion to Detroit 562 pharyngeal cells or nasopharyngeal colonization. Based on these results, we hypothesize lung epithelia may have an additional PspK ligand not present on pharyngeal cells. PspK has thus far been implicated in colonization and OM, but this data suggests PspK may also be important for adherence and pathogenesis within the lungs.

Biofilm production is a central characteristic of numerous bacterial species. For pneumococci, it is thought biofilm formation aids in colonization [39]. Biofilms also serve as a point of interaction between strains, facilitating genetic exchange of virulence factors and antibiotic resistances [40]. Previous studies have shown that polyamines are essential for biofilm formation by the plague-causing organism *Yersinia pestis* [34]. Additionally, biofilm formation by *Vibrio cholerae* was shown to increase when excess polyamines were added to growth media [35]. We examined changes in biofilm formation in both T4 and MNZ67 in the absence of a functional *pot* operon. However, these results were contrasting, with T4Δ*potD* having decreased biofilm formation and PIP01 having increased biofilm formation in comparison to WT. Polyamine acquisition is generally thought to aid in progression to biofilm, which seems to be the case for encapsulated pneumococci. Yet, in MNZ67 the inactivation of the polyamine transporter appears to enhance biofilm development.

Although we did not see an increase in pharyngeal cell adhesion in vitro (Fig 4A), we still wanted to examine in vivo nasopharyngeal colonization. Pneumococci must first colonize the nasopharynx before it can disseminate to other parts of the body and cause disease. It has been shown *potABCD* deletion significantly reduces the ability of encapsulated pneumococci to colonize the nasopharynx of a mouse [17], further supporting the claim that the pot operon is a good target for pneumococcal disease prevention. However, our results showed deletion of *potD* from MNZ67 did not reduce murine colonization or ascension from the nasopharynx to the middle ear. In fact, CFU recovered from mice infected with PIP01 were slightly higher than CFU recovered from mice infected with WT MNZ67 (Fig 6). Unlike encapsulated pneumococci, polyamine acquisition via the pot operon is not vital to successful colonization and disease development by NESp subgroup NCC1. This can be explained by the difference in biofilm formation, with reduced biofilm formation in encapsulated strain leading to reduced colonization and more biofilm in MNZ67 leading to slightly more colonization.

Middle ear infections are the most frequent reason for pediatric visits in the United States, and *S*. *pneumoniae* is a leading cause of OM [11]. Although the involvement of polyamine acquisition in other pneumococcal diseases has been explored, the role of polyamines in pneumococcal OM has not been determined. We therefore examined effects of *potD* deletion on T4 and MNZ67 associated OM in a chinchilla model. In MNZ67, *potD* deletion produced slight increases in recovered CFU, otic pathology, and biofilm formation. In contrast, deletion of *potD* in T4 significantly attenuated pathology, biofilm formation, and bacterial persistence in the middle ear. This data further supports our idea that polyamine acquisition by the pot operon is essential for pathogenesis of encapsulated pneumococci, but it is dispensable for pathogenesis of NCC1 NESp.

Together, the increased biofilm development, increased epithelial adhesion, increased colonization, and increased OM pathology reinforce that the absence of a functional *pot* operon in MNZ67 actually increases virulence. We propose two explanations for these results: 1) polyamine acquisition and metabolism negatively regulate virulence in the NESp, or 2) inactivation of the pot operon results in upregulation of polyamine biosynthetic pathways, thus overcompensating for the loss of polyamine acquisition and resulting in increased polyamine-associated virulence. In any case, we conclude polyamines are an integral component of pneumococcal virulence. Their role however, is altered between encapsulated and naturally occurring nonencapsulated strains. These finding exemplify the diversity amongst pneumococci and further elucidate the challenge of finding all-inclusive pneumococcal targets. Although our studies do not illustrate the pot operon to be a suitable target for reducing NESp virulence, our data does shed light on NCC1 protein regulation. Furthermore, our studies show NESp have unique mechanisms of virulence regulation that differ from encapsulated pneumococci. Together, our results illuminate the need to further investigate NESp virulence factors, virulence regulation, and metabolic processes.

## Supporting information

S1 Fig 1–3 Data. Datasets underlying Figs 1, 2, and 3. Protein levels were measured by indirect ELISA and pneumolysin function was determined by hemolysis assay. Triplicates were averaged, and percent production was calculated by dividing mutant values by WT/control values and multiplying by 100. Gene expression was determined by RT-qPCR. Change is expression was determined by the ΔΔCT method with *gyrA* serving as an internal control.
(PDF)

S2 Fig 4 Data. Dataset underlying Fig 4. Human epithelial cells were incubated with pneumococci to allow adherence or invasion. CFU was determined by plating on BA. Data is reported as log CFU.
(PDF)

S3 Fig 5 Data. Dataset underlying Fig 5. Biofilm formation was determined by crystal violet staining, alcohol solubilization, and OD_630_ analysis. OD readings and triplicate averages are reported here.
(PDF)

S4 Fig 6 Data. Dataset underlying Fig 6. Mice were intranasally infected with pneumococci and CFU were determined 5 days post infection. CFU counts for nasal samples and bullae were determined separately. Data is reported as log CFU.
(PDF)

S5 Fig 7 Data. Dataset underlying Fig 7. Chinchillas were infected in each bullae with pneumococci and CFU were determined 4 days post infection. Counts for the wash, exudate, and whole bullae were combined and data is reported as log CFU.
(PDF)

S6 Fig 8 Data. Dataset underlying Fig 8. Mice were intratracheally infected with pneumococci and CFU were determined 2 days post infection. Data is reported as log CFU.
(PDF)
