# Supplementary material for: Polyamine transporter potABCD is required for virulence of encapsulated but not nonencapsulated Streptococcus pneumoniae
Source: PLoS One. 2017 Jun 6;12(6):e0179159. doi: 10.1371/journal.pone.0179159 (PMC5460881; doi:10.1371/journal.pone.0179159)
Supplement: S1 Fig — Protein levels were measured by indirect ELISA and pneumolysin function was determined by hemolysis assay. Triplicates were averaged, and percent production was calculated by dividing mutant values by WT/control values and multiplying by 100. Gene expression was determined by RT-qPCR. Change is expression was determined by the ΔΔCT method with gyrA serving as an internal control. (PDF) [file pone.0179159.s001.pdf]

Ply Expression Determined by ELISA

Sample #	MNZ67	PIP01
1	0.331	0.209
1	0.329	0.211
1	0.436	0.21
2	0.358	0.25
2	0.353	0.244
2	0.324	0.245
3	0.341	0.302
3	0.394	0.331
3	0.371	0.308
	Average	
1	0.365333333	0.256666667
2	0.345	0.246333333
3	0.368666667	0.313666667

Percent Expression Releative to WT

67	PIP01
100	70.25547445
100	71.4009662
100	85.08137

Hemolysis Assay

	Triton X-114	MNZ67	PIP01
1	0.475	0.468	
1	0.441	0.459	
1	0.461	0.477	
2	0.459	0.46	
2	0.453	0.452	
2	0.459	0.445	
3	0.443	0.435	
3	0.427	0.469	
3	0.462	0.441	
Average			
1	0.459	0.456222222	
2	0.457	0.452333333	
3	0.444	0.448333333	

Percent Hemolysis Relative to + Control

Triton X-114	MNZ67	PIP01
100	102	57.22
100	99	65
100	101	67

PspK Levels Determined by ELISA

Sample	MNZ67	PIP01
1	0.27	0.8
1	0.263	0.828
1	0.259	0.835
2	0.265	0.56
2	0.292	0.542
2	0.271	0.554
3	0.21	0.75
3	0.227	0.8
3	0.223	0.76

	Average	
1	0.264	0.821
2	0.276	0.552
3	0.22	0.77

Percent Relative to WT

67	PIP01
100	200
100	310.98
100	350

CT Values and	GyrA Rep-1	GyrA Rep-2	PspK Rep-1	Pspk Rep-1
Sample	24.3	23.5	26.1	28.6
67- Exp 1	23.8	23.8	25.9	27.3
PIP01-Exp1	27.3	26.7	28.3	27.2
67-Exp2	23.9	24.6	24.1	24.8
PIP01-Exp2	25.2	26.8	29.5	29.8
67-Exp3	24.1	23.9	27.1	27.6
PIp01-Exp3				

Sample	GyrA Average	PspK Average	ΔCt	ΔΔCt	2-ΔΔCT
67- Exp 1	23.9	27.35	3.45	0	1
PIP01-Exp1	23.8	26.6	2.8	-0.65	1.569168196
67-Exp2	27	27.75	0.75	0	1
PIP01-Exp2	24.25	24.45	0.2	-0.55	1.464085696
67-Exp3	26	29.65	3.65	0	1
PIp01-Exp3	24	27.35	3.35	-0.3	1.231144413

PspK After *potD* Complementation

	MNZ67	PIP01	PIP02
1	0.235	0.315	0.22
1	0.246	0.313	0.219
1	0.29	0.32	0.209
2	0.221	0.37	0.212
2	0.276	0.365	0.232
2	0.298	0.345	0.235
3	0.254	0.295	0.225
3	0.298	0.31	0.235
3	0.24	0.283	0.224

Average

1	0.257	0.324	0.223444444
2	0.265	0.36	0.226333333
3	0.264	0.296	0.228

Percent Relative to WT

MNZ67	PIP01	PIP02
100	126.070039	86.9433636
100	112.1212121	85.4088
100	135.8490566	86.364

Ply After *potD* Complementation

	MNZ67	PIP01	PIP02
	0.345	0.258	0.5
	0.339	0.225	0.59
	0.327	0.282	0.625
	0.312	0.25	0.462
	0.332	0.249	0.447
	0.31	0.3	0.477
	0.319	0.252	0.412
	0.321	0.31	0.405
	0.356	0.325	0.409

Average

1	0.337	0.27233333	0.48077778
2	0.318	0.26633333	0.462
3	0.332	0.295666667	0.408666667

Percent Relative to WT		MNZ67	PIP01	PIP02
		100	80.811078	123.0923695
		100	83.752605	145.283019
		100	89.0562249	142.664029
