# Supplementary material for: Polyamine transporter potABCD is required for virulence of encapsulated but not nonencapsulated Streptococcus pneumoniae
Source: PLoS One. 2017 Jun 6;12(6):e0179159. doi: 10.1371/journal.pone.0179159 (PMC5460881; doi:10.1371/journal.pone.0179159)
Supplement: S2 Fig — Human epithelial cells were incubated with pneumococci to allow adherence or invasion. CFU was determined by plating on BA. Data is reported as log CFU. (PDF) [file pone.0179159.s002.pdf]

Epithelial Cell Adhesion and Invasion

A549 Adhesior	MNZ67 CFU	PIP01 CFU	Detroit 562 Ad	MNZ67 CFU	PIP01 CFU
1	1.91E+04	3.23E+04	1	7.30E+04	9.53E+04
1	9.29E+03	1.44E+04	1	1.20E+05	5.48E+04
1	6.13E+03	2.92E+04	1	1.12E+05	1.17E+05
2	1.71E+04	2.80E+04	2	1.01E+05	1.46E+05
2	3.33E+04	1.43E+04	2	5.75E+04	1.21E+05
2	1.95E+04	2.65E+04	2	7.75E+04	1.53E+05
3	1.06E+04	2.81E+04	3	1.15E+05	6.85E+04
3	1.33E+04	3.76E+04	3	7.25E+04	4.76E+04
3	1.75E+04	2.12E+04	3	8.90E+04	9.10E+04

	log CFU	log CFU		log CFU	log CFU
	4.28115841	4.50903442		4.86332286	4.97893337
	3.9682293	4.1568519		5.07736791	4.73838412
	3.78760222	4.46556724		5.04921802	5.0687979
	4.23339111	4.44715028		5.00432137	5.16524433
	4.52211806	4.15395199		4.75966784	5.08357428
	4.28987256	4.42279496		4.8893017	5.18346915
	4.02541893	4.44865995		5.05880549	4.83550033
	4.12232934	4.57564961		4.86033801	4.67756133
	4.24290278	4.32643828		4.94939001	4.95911297

Epithelial Cell Adhesion and Invasion

A549 Invasion	MNZ67 CFU	PIP01 CFU	Detroit 562 In	MNZ67 CFU	PIP01 CFU
1	4.39E+02	3.50E+03	1	2.22E+03	8.20E+02
1	2.87E+02	2.82E+03	1	1.05E+03	1.49E+03
1	8.35E+02	2.15E+03	1	1.60E+03	1.48E+03
2	1.05E+02	6.81E+02	2	8.56E+02	1.27E+03
2	1.62E+03	2.57E+03	2	3.15E+03	8.65E+02
2	8.56E+02	1.64E+02	2	1.17E+03	9.67E+02
3	1.11E+03	9.51E+02	3	8.56E+02	1.59E+03
3	1.67E+03	1.51E+03	3	1.91E+03	2.92E+03
3	2.14E+03	1.75E+03	3	1.70E+03	1.33E+03

log CFU	log CFU	log CFU	log CFU
2.64254464	3.54392284	3.34642535	2.91381385
2.45824492	3.45033072	3.01951923	3.17172645
2.92184768	3.33236371	3.20431944	3.17128755
2.01953168	2.83293214	2.93236112	3.10473287
3.21069031	3.41034357	3.49828712	2.937247
2.93236112	2.21432185	3.06681028	2.98554547
3.04582154	2.97812389	2.93236112	3.20105556
3.22353618	3.17843878	3.28069216	3.46475177
3.32958458	3.24251658	3.22953825	3.12466722
