# Supplementary material for: Polyamine transporter potABCD is required for virulence of encapsulated but not nonencapsulated Streptococcus pneumoniae
Source: PLoS One. 2017 Jun 6;12(6):e0179159. doi: 10.1371/journal.pone.0179159 (PMC5460881; doi:10.1371/journal.pone.0179159)
Supplement: S3 Fig — Biofilm formation was determined by crystal violet staining, alcohol solubilization, and OD630 analysis. OD readings and triplicate averages are reported here. (PDF) [file pone.0179159.s003.pdf]

CRYSTAL VIOLET BIOFILM STAINING READ AT OD620

REPLICATES	67	PIP01	T4	T4ΔpotD
1	0.224	0.399	0.309	0.158
1	0.251	0.409	0.322	0.151
1	0.277	0.428	0.32	0.171
2	0.172	0.184	0.35	0.2
2	0.201	0.269	0.357	0.209
2	0.236	0.304	0.337	0.224
3	0.175	0.299	0.312	0.23
3	0.209	0.397	0.321	0.236
3	0.296	0.401	0.333	0.212
4	0.187	0.3	0.345	0.1
4	0.216	0.368	0.358	0.105
4	0.242	0.31	0.377	0.107
5			0.372	0.225
5			0.359	0.224
5			0.409	0.262
6			0.371	0.2
6			0.379	0.21
6			0.392	0.205
AVERAGE				
1	0.250666667	0.412	0.317	0.1905
2	0.203	0.252333333	0.348	0.211
3	0.226666667	0.365666667	0.322	0.226
4	0.215	0.326	0.36	0.104
5			0.38	0.237
6			0.38066667	0.205
