# Supplementary material for: Polyamine transporter potABCD is required for virulence of encapsulated but not nonencapsulated Streptococcus pneumoniae
Source: PLoS One. 2017 Jun 6;12(6):e0179159. doi: 10.1371/journal.pone.0179159 (PMC5460881; doi:10.1371/journal.pone.0179159)
Supplement: S4 Fig — Mice were intranasally infected with pneumococci and CFU were determined 5 days post infection. CFU counts for nasal samples and bullae were determined separately. Data is reported as log CFU. (PDF) [file pone.0179159.s004.pdf]

COLONIZAION AND MIDDLE EAR ASCENSION DATA
W= NASOPHRYNGEAL WASH
NP= NASOPHARYNGEAL TISSUE
B= BULLAE

	67	PIP01	LOG 67	LOGPIP01
W+NP	4.50E+03	9.50E+03	3.653212514	3.977723605
W+NP	2.00E+02	7.50E+03	2.301029996	3.875061263
W+NP	3.08E+03	7.75E+03	3.488550717	3.889301703
W+NP	1.00E+03	3.25E+02	3	2.511883361
W+NP	6.75E+03	1.88E+03	3.829303773	3.273001272
B1+B2	1.50E+02	8.25E+02	2.176091259	2.916453949
B1+B2	5.00E+01	2.50E+02	1.698970004	2.397940009
B1+B2	4.50E+02	2.50E+02	2.653212514	2.397940009
B1+B2	7.50E+01	6.75E+02	1.875061263	2.829303773
B1+B2	1.50E+02	7.50E+01	2.176091259	1.875061263

	67	PIP01	LOG 67	LOG PIP01
W+NP	6.00E+02	5.00E+01	2.77815125	1.698970004
W+NP	5.00E+01	9.50E+02	1.698970004	2.977723605
W+NP	4.00E+03	6.75E+02	3.602059991	2.829303773
W+NP	5.00E+01	7.00E+03	1.698970004	3.84509804
W+NP	5.00E+01	6.00E+02	1.698970004	2.77815125
B1+B2	7.50E+01	5.00E+01	1.875061263	1.698970004
B1+B2	1.50E+02	5.00E+02	2.176091259	2.698970004
B1+B2	1.75E+02	7.50E+01	2.243038049	1.875061263
B1+B2	1.50E+02	1.50E+03	2.176091259	3.176091259
B1+B2	1.00E+02	5.00E+01	2	1.698970004
