# Supplementary material for: Polyamine transporter potABCD is required for virulence of encapsulated but not nonencapsulated Streptococcus pneumoniae
Source: PLoS One. 2017 Jun 6;12(6):e0179159. doi: 10.1371/journal.pone.0179159 (PMC5460881; doi:10.1371/journal.pone.0179159)
Supplement: S5 Fig — Chinchillas were infected in each bullae with pneumococci and CFU were determined 4 days post infection. Counts for the wash, exudate, and whole bullae were combined and data is reported as log CFU. (PDF) [file pone.0179159.s005.pdf]

B1-4 are from experiment 1. B5-10 are from experiment 2
E= Exudate, W= wash, B=Whole Bullae

	PP01	SUM PER BULLA	LOG CFU per E
E1	1.13E+06	B1	
W1	5.25E+05	2.33E+06	6.367355921
B1	6.75E+05		
E2	5.00E+06	B2	
W2	1.13E+06	7.18E+06	6.856124444
B2	1.05E+06	B3	
E3	5.00E+01	1.25E+05	5.09725731
W3	5.00E+01	B4	
B3	1.25E+05	8.26E+04	4.916980047
E4	8.25E+04	B5	
W4	5.00E+01	9.33E+04	4.969997999
B4	5.00E+01	B6	
E5	5.00E+01	2.01E+04	4.303196057
W5	7.75E+02	B7	
B5	9.25E+04	9.04E+04	4.956384563
E6	2.00E+04	B8	
W6	5.00E+01	1.37E+05	5.136543009
B6	5.00E+01	B9	
E7	8.24E+04	4.82E+04	4.683236212
W7	4.53E+03	B10	
B7	3.52E+03	2.38E+05	5.376412697
E8	9.44E+02		
W8	1.00E+03		
B8	1.35E+05		
E9	3.21E+04		
W9	8.14E+03		
B9	8.00E+03		
E10	8.90E+04		
W10	7.18E+04		
B10	7.71E+04		

B1-4 are from experiment 1. B5-10 are from experiment 2
E= Exudate, W= wash, B=Whole Bullae

ɜulla	MNZ67		SUM PER BULLA	LOG CFU per E
E1	8.90E+04			
W1	7.18E+04	B1	1.85E+05	5.267406419
B1	2.43E+04			
E2	8.75E+03			
W2	1.63E+03	B2	2.99E+04	4.475380593
B2	1.95E+04			
E3	1.99E+04	B3	1.50E+05	5.176322821
W3	5.18E+03			
B3	1.25E+05			
E4	7.95E+03	B4	9.45E+04	4.975321497
W4	3.20E+04			
B4	5.45E+04			
E5	1.75E+04	B5	8.82E+04	4.945320841
W5	2.05E+04			
B5	5.02E+04			
E6	1.94E+04			
W6	7.65E+01	B6	2.05E+04	4.31126527
B6	1.00E+03			
E7	2.00E+03			
W7	1.26E+04	B7	1.63E+04	4.211267418
B7	1.72E+03			
E8	1.37E+03			
W8	1.00E+04	B8	3.04E+04	4.482448367
B8	1.90E+04			
E9	6.00E+04			
W9	1.33E+04	B9	7.37E+04	4.867467488
B9	4.50E+02			
E10	3.21E+04			
W10	5.50E+01	B10	3.22E+04	4.507667007
B10	5.00E+01			

B1-4 are from experiment 1. B5-10 are from experiment 2
E= Exudate, W= wash, B=Whole Bullae

Bulla	T4		SUM PER BULLA	LOG CFU per B
E1	1.75E+05	B1	2.38E+05	5.375663614
W1	6.25E+04			
B1	0.00E+00			
E2	5.24E+04	B2	7.07E+06	6.849259672
W2	7.00E+06			
B2	1.50E+04			
E3	6.00E+04	B3	7.57E+04	4.87909588
W3	1.50E+04			
B3	7.00E+02			
E4	2.00E+02	B4	7.01E+06	6.845544513
W4	7.00E+03			
B4	7.00E+06			
E5	1.03E+07	B5	2.41E+07	7.382287266
W5	1.50E+04			
B5	1.38E+07	B6	2.55E+07	7.407135862
E6	3.50E+04			
W6	1.70E+07			
B6	8.50E+06			
E7	4.00E+05	B7	4.05E+06	6.607455023
W7	3.50E+06			
B7	1.50E+05			
E8	1.90E+05	B8	3.76E+06	6.575187845
W8	4.70E+05			
B8	3.10E+06			

B1-4 are from experiment 1. B5-10 are from experiment 2
E= Exudate, W= wash, B=Whole Bullae

Bulla	T4(ΔpotD)		SUM PER BULLA	LOG CFU per B
E1	5.00E+01	B1	1.50E+02	2.176091259
W1	5.00E+01			
B1	5.00E+01			
E2	5.00E+01	B2	1.25E+02	2.096910013
W2	2.50E+01			
B2	5.00E+01			
E3	2.50E+01	B3	1.16E+04	4.063521
W3	1.15E+04			
B3	5.00E+01			
E4	5.00E+01	B4	1.26E+04	4.100370545
W4	1.25E+04			
B4	5.00E+01			
E5	3.75E+05	B5	3.75E+05	5.574147064
W5	5.00E+01			
B5	5.00E+01	B6	2.51E+04	4.399673721
E6	2.50E+04			
W6	5.00E+01	B7	5.51E+04	4.741151599
B6	5.00E+01			
E7	5.50E+04	B8	2.01E+04	4.303196057
W7	5.00E+01			
B7	5.00E+01			
E8	2.00E+04	B9	6.57E+03	3.81756537
W8	5.00E+01			
B8	5.00E+01			
E9	6.70E+02			
W9	7.50E+02	B10	5.25E+03	3.720159303
B9	5.15E+03			
E10	1.00E+03			
W10	4.00E+03			
B10	2.50E+02			

B1-4 are from experiment 1. B5-10 are from experiment 2
E= Exudate, W= wash, B=Whole Bullae

3ulla
