# Supplementary material for: Polyamine transporter potABCD is required for virulence of encapsulated but not nonencapsulated Streptococcus pneumoniae
Source: PLoS One. 2017 Jun 6;12(6):e0179159. doi: 10.1371/journal.pone.0179159 (PMC5460881; doi:10.1371/journal.pone.0179159)
Supplement: S6 Fig — Mice were intratracheally infected with pneumococci and CFU were determined 2 days post infection. Data is reported as log CFU. (PDF) [file pone.0179159.s006.pdf]

LUNGS HARVESTED 2 DAYS POST INFECTION

	67	PIP01
1	1.00E+02	2.90E+03
2	3.00E+02	3.40E+03
3	1.00E+02	2.40E+03
4	3.00E+02	2.50E+03
5	2.00E+02	1.50E+03

Log CFU per Mouse	
2	3.462397998
2.477121255	3.531478917
2	3.380211242
2.477121255	3.397940009
2.301029996	3.176091259
